# Suicide attempts in the adult Mexican population: an analysis of sociodemographic characteristics and associated factors

**DOI:** 10.1590/1980-549720240014

**Published:** 2024-03-18

**Authors:** Claudio Alberto Davila-Cervantes, Marisol Luna-Contreras

**Affiliations:** I Latin American Faculty of Social Sciences, Mexico.

**Keywords:** Suicide, Suicide attempt, Mexico, Associated factors, Adults, Suicídio, Tentativa de suicídio, México, Fatores associados, Adultos

## Abstract

**Objective:**

Suicide is the culmination of a process or continuum known as suicidal behavior that proceeds from ideation and planning to attempt. The objective was to estimate the prevalence of suicide attempts in the adult Mexican population and to analyze their main associated factors.

**Methods:**

We conducted an observational, cross-sectional, and descriptive study with information from the National Health and Nutrition Survey (2018). Self-reported lifetime suicide attempt was used in the analysis. We analyzed depression, obesity, tobacco smoking, and alcohol consumption as suicide attempt-associated factors using a multivariate logistic regression model.

**Results:**

The prevalence of adult suicide attempt was 2.0% (95%CI 1.8–2.2) and it was higher among women (2.4%; 95%CI 2.2–2.8) and young people (2.9%; 95%CI 2.4–3.4). Low education (OR=1.6; 95%CI 1.2–2.2), being single (OR=1.3; 95%CI 1.0–1.6), having obesity (OR=1.4; 95%CI 1.1–1.8), consumption of alcohol (OR=2.4; 95%CI 1.7–3.4) or tobacco smoking (OR=1.8; 95%CI 1.4–2.4), and having strong symptoms of depression (OR=10.1; 95%CI 6.2–16.3) were associated with a higher prevalence of suicide attempts.

**Conclusion:**

These results help better understand suicidal behavior in Mexico and identify the factors that increase the likelihood of suicide attempts, which is essential to help reduce suicide mortality. This research is crucial for developing early interventions and prevention programs aimed at reducing suicide's public health burden.

## RESUMO

**Objetivo:**

O suicídio é o resultado de um processo ou continuidade conhecido como comportamento suicida, que parte da ideação e planejamento até a tentativa. O objetivo foi estimar a prevalência de tentativa de suicídio na população adulta mexicana e analisar seus principais fatores associados.

**Métodos:**

Realizamos um estudo observacional, transversal e descritivo com informações da Pesquisa Nacional de Saúde e Nutrição (2018). Na análise, foi utilizado o relato de tentativa de suicídio ao longo da vida. Analisamos a depressão, a obesidade, o tabagismo e o consumo de álcool como fatores associados à tentativa de suicídio, usando um modelo de regressão logística multivariada.

**Resultados:**

A prevalência de tentativas de suicídio em adultos foi de 2,0% (intervalo de confiança de 95% — IC95% 1,8-2,2); foi mais alta entre as mulheres (2,4%; IC95% 2,2–2,8) e entre os jovens (2,9%; IC95% 2,4–3,4). Baixa educação (*odds ratio* — OR=1.6; IC95% 1.2–2.2), estado civil solteiro (OR=1.3; IC95% 1.0–1.6), obesidade (OR=1.4; IC95% 1.1–1.8), consumo de álcool (OR=2.4; IC95% 1.7–3.4) ou tabaco (OR=1.8; IC95% 1.4–2.4) e sintomas fortes de depressão (OR=10.1; IC95% 6.2–16.3) estão associados a uma maior prevalência de tentativas de suicídio.

**Conclusão:**

Esses resultados ajudam a entender melhor o comportamento suicida no México e a identificar os fatores que aumentam a probabilidade de tentativas de suicídio, o que é essencial para ajudar a reduzir a mortalidade por essa causa. Esta pesquisa é crucial para o desenvolvimento de intervenções precoces e programas de prevenção com o objetivo de reduzir o ônus de saúde pública do suicídio.

## INTRODUCTION

According to the World Health Organization (WHO), in 2019, 703,000 people died by suicide worldwide, and three-quarters of these deaths occurred in low- and middle-income countries^1^. Each death by suicide is an individual tragedy that indirectly affects 135 people^2^. For this reason, suicide is considered a priority global public health problem, affecting all ages^3^.

Suicide is the culmination of a process from ideation and planning to attempts^4^. A suicide attempt is defined as a self-destructive act aimed at taking one's own life^5^, with one suicide death for every estimated 20 attempts^1^. The prevalence of lifetime suicide attempt ranges from 3 to 5%^6^. Life expectancy is shortened by 18 years in men and 11 years in women after a suicide attempt at 20 years of age^7^.

In Mexico, various studies have addressed suicide attempts, reporting a prevalence of 3.75% between 18 and 29 years of age and of 2.61% between 30 and 65 years of age^8^. Other studies estimated that the prevalence of lifetime suicide attempt is between 2.5 and 4.3%^9^. The annual prevalence of suicide attempts in the adult Mexican population ranges from 0.62 to 0.90%^8,10^.

Suicide attempt is associated with various factors^11^. Evidence shows that a prior suicide attempt is the greatest associated factor for a subsequent attempt^1^, and up to three-quarters of people who have attempted suicide are at risk of a new attempt^12^. Previous studies done in different contexts found that substance use (alcohol, tobacco, and drugs)^13^, depression^11^, sex^14^, negative life events (violence, loss of loved ones, or marital problems)^15,16^, and socioeconomic factors (educational level, unemployment, or poverty)^14,17^ are associated with suicide attempts.

Research into suicide attempts and their prevalence and associated factors in Mexico is essential for their high psychological burden, repercussions, injuries, and their close relationship with completed suicide^9^, which has been increasing in Mexico^10^. Thus, the objective of this study was to estimate the prevalence of suicide attempts in the adult Mexican population and to analyze their main associated factors. To the best of our knowledge, no other study has analyzed the recent prevalence of suicide attempt and its associated factors in the adult population of Mexico. These results will help better understand suicidal behavior in Mexico^10^ and to identify the factors that increase the likelihood of suicide attempts^18^. This research is crucial for developing early interventions and prevention programs aimed at reducing the public health burden of suicide^14^.

## METHODS

An observational, cross-sectional study was conducted based on secondary data from Mexico's National Health and Nutrition Survey 2018–2019 (ENSANUT-2018) with population aged 20 years or older living in Mexico at the time of the survey. One objective of ENSANUT-2018 was to gather information on the most relevant health and nutrition indicators of the Mexican population^19^. The microdata used come from the Adult Health Questionnaire (CSA). The data collection period was from July 30, 2018, to June 28, 2019. The survey's sampling design has been published elsewhere^19^. The sample size of the CSA was 43,070 interviews, but the estimates were made using information from 42,269 individuals, which correspond to those who answered yes or no to the question of lifetime suicide attempt and that contained complete information about the sociodemographic characteristics and associated factors. We used the "listwise deletion" procedure that is a method considered valid when dealing with Missing Completely at Random data. The p-value of Little's test was 0.763. The unit of analysis was Mexican adults aged 20 years or older who have information indicating whether they have ever attempted to take their own life.

### Dependent and explanatory variables

The dependent variable was lifetime suicide attempt, which was measured using the question: Have you ever intentionally injured, cut, poisoned, or harmed yourself to commit suicide? The answers "Yes, once" or "Yes, twice or more times" were classified as "Yes" (1) and the answer "Never" (0). The explanatory variables were as follows: sociodemographic characteristics such as sex, age, education, and marital status (Table 1) and associated factors such as depression, obesity, tobacco smoking, and alcohol consumption. Depression was estimated using a depressive symptoms index determined by performing a principal component analysis (PCA). The PCA was performed with a matrix of polychoric correlations. The first principal component was retained because it explained 80.2% of the variance and was the only one with an eigenvalue ³1. The Kaiser-Meyer-Olkin index was 89.1% (sampling adequacy), and Cronbach's alpha was 0.89 (high internal consistency). We divided the index into three groups (Table 1).

**Table 1:** Explanatory variables' operationalization.

Characteristics	Variables	Operationalization	Observations
Sociodemographic	Sex	0: Females	Based on self-reported at the time of the survey
		1: Males	
	Age (years)	0: 20–29	Based on self-reported at the time of the survey
		1: 30–39	
		2: 40–49	
		3: 50 or more	
	Education	Never attended school or incomplete elementary school	Based on self-reported educational stage and grade at the time of the survey
		0: Never attended school or incomplete elementary school	
		1: Completed elementary school	
		2: Completed middle school	
		3: Completed high school or higher	
	Marital status	0: Single	Based on self-reported at the time of the survey
		1: Married or cohabiting	
Associated factors	Obesity	0: No	Based on the answer to the question "Have a doctor/dietician/nutritionist ever told you that you have had or currently have obesity?"
		1: Yes	
	Tobacco smoking	0: No	Based on the answer to the question "Have you smoked at least 100 cigarettes (5 packs) of tobacco in your entire life?"
		1: Yes	
	Alcohol consumption	0: Have never consumed	Based on self-reported consumption in the last month
		1: Do not consume	
		2: Do consume	
	Depression symptoms	0: Without symptoms	Based on the responses from the depressive symptoms module of the survey.
		1: Mild or moderate symptoms	
		3: Strong symptoms	

### Statistical analysis

We calculated the prevalence rate of lifetime suicide attempts among Mexican adults at the national level. We estimated the prevalence of lifetime suicide attempt by sociodemographic characteristics and associated factors. The corresponding 95% confidence intervals (95%CIs) were also calculated. The bivariate analysis of the prevalence of suicide attempts for the explanatory variables was performed using the Pearson chi-squared tests to assess whether the prevalence of suicide attempts and each of the explanatory variables were significantly different. A multivariate logistic regression model (LRM) was estimated to identify the characteristics that increased the likelihood of attempting suicide. We included an interaction between depression symptoms and sex to observe how the depression effects on suicide attempt differ between men and women. We used the "full" method, including all independent variables in the model, to mitigate selection bias and ensure accurate standard errors and p-values^20,21^. The results of the LRM were interpreted using adjusted odds ratios (ORs). Although OR is not the most suitable measure for epidemiological studies, it is commonly used to enable comparisons with other studies^22,23^.

To provide a more comprehensive interpretation of the variables included in the interaction, we present the results in terms of probabilities and marginal effects. This approach involved maintaining the remaining variables at their mean values when calculating these probabilities and effects. This allows a more nuanced understanding of the relationship between the variables.

The goodness-of-fit was assessed using the Hosmer–Lemeshow test, in addition to inquiring about the existence of influential data. The quality of the model was analyzed via a classification table. We also verified that there was no collinearity among the explanatory variables included in the LRM, using the variance inflation factor (VIF), and found that the VIF was less than 10 for all explanatory variables. The information was processed using Stata/MP version 15.1, considering the sampling design. Thus, we considered the primary sampling units, sampling strata, and sampling weights in all estimations. The coefficient of variation was lower than 30%. Therefore, the level of precision of the estimates was at least moderate. We used aggregated secondary public data that guaranteed the confidentiality of the subjects under study; thus, there are no ethical conflicts. All the databases used are accessible to the public.

## RESULTS

Women accounted for 54.6% of all participants, and 35% of the participants were 50 years of age or older. Most interviewees were married or cohabiting (63.9%), and nearly two-thirds had at least completed secondary education. Regarding tobacco smoking, 28.2% of the adults were smokers at some point, and 37% had never consumed alcohol. Additionally, 23% interviewees had been diagnosed with obesity by a healthcare provider, and 16.3% had strong depressive symptoms (Table 2).

**Table 2:** Percentage distribution of adults by sociodemographic characteristics and associated factors in 2018 in Mexico.

Characteristics		n	%	95%CI
Sex				
	Males	19,122	45.4	(44.7–46.1)
	Females	23,147	54.6	(53.9–55.4)
Age (years)				
	20–29	8,686	23.6	(22.9–24.2)
	30–39	9,543	21.0	(20.4–21.6)
	40–49	8,723	20.0	(19.5–20.6)
	50 or more	15,317	35.4	(34.7–36.2)
Education				
	Never attended school or incomplete elementary school	7,998	16.9	(16.3–17.4)
	Completed elementary school	7,447	17.6	(17.0–18.1)
	Completed middle school	12,613	29.1	(28.4–29.8)
	Completed high school or higher	14,211	36.5	(35.7–37.3)
Marital status				
	Single	16,134	36.1	(35.4–36.8)
	Married or cohabiting	26,135	63.9	(63.2–64.6)
Obesity				
	No	32,374	77.2	(76.5–77.8)
	Yes	9,895	22.9	(22.2–23.5)
Tobacco smoking				
	No	30,278	71.8	(71.1–72.5)
	Yes	11,991	28.2	(27.5–28.9)
Alcohol consumption				
	Have never consumed	14,767	36.6	(35.9–37.4)
	Do not consume	17,168	40.4	(39.6–41.2)
	Do consume	10,334	23.0	(22.3–23.6)
Depression symptoms				
	Without symptoms	18,272	44.9	(44.1–45.7)
	Mild or moderate symptoms	16,609	38.8	(38.1–39.6)
	Strong symptoms	7,388	16.3	(15.7–16.8)

n: 42,269; 95%CI: 95% confidence interval.

The national prevalence of suicide attempt was 2.0% among adults and was higher in women (2.4%) than in men (1.5%). The percentage of suicide attempts was higher in young adults aged 20–29 years (2.9%) than in all other age groups. The prevalence of suicide attempt was higher among single adults (2.5%) than among their married or cohabiting counterparts (1.7%). Adults with completed high school or higher (1.6%) had a lower prevalence than those with completed middle school (2.5%) (Table 3).

**Table 3:** Prevalence of lifetime suicide attempt by sociodemographic characteristics and associated factors in 2018 in Mexico.

Characteristics		n	%	95%CI
Sex*				
	Males	290	1.4	(1.1– 1.6)
	Females	605	2.4	(2.2–2.8)
Age (years)†				
	20–29	267	2.9	(2.4–3.4)
	30–39	214	1.9	(1.6–2.3)
	40–49	195	2.0	(1.6–2.5)
	50 or above	219	1.3	(1.1–1.6)
Education‡				
	Never attended school or incomplete elementary school	145	1.6	(1.3–2.1)
	Completed elementary school	184	2.2	(1.8–2.6)
	Completed middle school	318	2.5	(2.1–3.0)
	Completed high school or higher	248	1.6	(1.3–1.9)
Marital status†				
	Single	455	2.5	(2.1–2.8)
	Married or cohabiting	440	1.7	(1.4–1.9)
Obesity†				
	No	602	1.7	(1.5–1.9)
	Yes	293	2.9	(2.4–3.4)
Tobacco smoking†				
	No	523	1.6	(1.4–1.8)
	Yes	371	2.9	(2.5–3.3)
Alcohol consumption†				
	Have never consumed	204	1.2	(1.0–1.5)
	Do not consume	414	2.2	(1.9–2.5)
	Do consume	264	2.7	(2.2–3.3)
Depression symptoms†				
	Without symptoms	143	0.8	(0.6–1.0)
	Mild or moderate symptoms	311	1.7	(1.4–2.0)
	Strong symptoms	441	5.8	(5.1–6.7)

n: 42,269; 95%CI: 95% confidence interval.

* Significance of the χ^2^ independence test: p-value<0.05

† p-value<0.0001

‡ p-value<0.01.

Considering the associated factors, the prevalence of suicide attempts was higher among adults who were or had previously been diagnosed with obesity (2.9%) than among those who were not and had never been obese (1.7%) (Table 3). Adult tobacco smokers (2.9%), those who had consumed alcohol but not in the last 30 days before the survey (2.2%), and those who consumed alcohol (2.7%) showed higher prevalence rates of suicide attempts than their counterparts without these habits, respectively. Adults with strong depressive symptoms (5.8%) had the highest prevalence of suicide attempts. Both sociodemographic characteristics and associated factors were significantly associated with suicide attempts (p-values <0.05 of the chi-squared tests).

The results of the LRM are outlined in Table 4. As shown, being older is a protective factor against suicide attempts; younger adults (aged 20–29 years) were 2.8 times more likely to attempt suicide at some point in their lives than adults aged 50 years or above when controlling for other characteristics, a likeliness that decreases to 1.7 times for the age groups from 30 to 39 years and from 40 to 49 years. Adults with completed elementary or middle school education were 1.6 times more likely to have ever attempted suicide than adults with a high school diploma or a higher level of education. Single adults had a higher likelihood of suicide attempts (OR=1.3) than those who were married or cohabiting.

**Table 4:** Results from logistic regression model for lifetime suicide attempt among adults in 2018 in Mexico.

Characteristics		OR	95%CI
Sex				
	Males*		
	Females	3.6†	(2.1–6.1)
Age (years)				
	20–29	2.8†	(1.9–4.0)
	30–39	1.7‡	(1.2–2.4)
	40–49	1.7†	(1.2–2.5)
	50 or above†		
Education				
	Never attended school or incomplete elementary school	1.4	(0.9–2.1)
	Completed elementary school	1.6†	(1.2–2.2)
	Completed middle school	1.6†	(1.2–2.1)
	Completed high school or higher*		
Marital status				
	Single	1.3§	(1–1.6)
	Married or cohabiting*		
Obesity				
	No*		
	Yes	1.4‡	(1.1–1.8)
Tobacco smoking				
	No*		
	Yes	1.8†	(1.4–2.4)
Alcohol consumption				
	Have never consumed*		
	Do not consume	1.9†	(1.5–2.6)
	Do consume	2.4†	(1.7–3.4)
Depression symptoms				
	Without symptoms*		
	Mild or moderate symptoms	2.9†	(1.8–4.7)
	Strong symptoms	10.1†	(6.2–16.3)
Sex and depression symptoms				
	Male without symptoms*		
	Female with mild or moderate symptoms	0.51§	(0.3–1)
	Female with strong symptoms	0.54§	(0.3–1)

n: 42,269; Goodness-of-fit: p-value Archer–Lemeshow F test=0.553; p-value test of specification link test for single-equation models hat » 0.00 and hatsq=0.060; 71.7% of correct classified cases.

* Reference category

† p-value<0.000

‡ p-value<0.01

§ p-value<0.05.

After controlling for sociodemographic characteristics and depressive symptoms, associated factors such as having suffered or suffering from obesity (OR=1.4), having consumed alcohol but not in the 30 days before the interview (OR=1.9 times), currently consuming alcohol (OR=2.4), and being a tobacco smoker at some point in life (OR=1.8) increased the likelihood of suicide attempts among Mexican adults in comparison with their peers without these characteristics (Table 4).

Due to the incorporation of an interaction variable between sex and depressive symptoms, the OR for sex reflects the impact of being female, while accounting for the reference category of depressive symptoms (without symptoms), on the probability of lifetime suicide attempt. Specifically, women without depressive symptoms exhibit a 3.6 higher likelihood of lifetime suicide attempt compared with men. The OR corresponding to depressive symptoms sheds light on the impact of depression, accounting for the reference category of sex, which in this context is men. Notably, the increase in depressive symptomatology among men positively correlates with an escalated propensity for lifetime suicide attempts. For instance, men manifesting mild or moderate depressive symptoms were 2.9 times more prone to a lifetime suicide attempt compared with those without depressive symptoms. This propensity surges to 10.1 times for men with severe depressive symptoms. Regarding the OR pertinent to the interaction, the sex disparities in lifetime suicide attempts show statistical significance and fluctuate in consonance with the severity of depressive symptomatology. The sex disparities in lifetime suicide attempts, favoring females, diminish by a factor of 2.0 (1/0.51) for individuals with mild or moderate depressive symptoms and by a factor of 1.8 (1/0.54) for those with strong depressive symptoms, relative to individuals without depressive symptoms.

A more detailed and clear interpretation of the results of the interaction between sex and depressive symptoms can be observed using the probabilities and marginal effects obtained for lifetime suicide attempts (Table 5). The probability that an adult woman has attempted suicide at some time in her life is 1.0% higher than that of an adult man. The relationship between suicide attempts and depressive symptoms is directly proportional among Mexican adults because the probability of attempting suicide increases with the rise in depressive symptoms. Accordingly, the probability of adults with mild or moderate depressive symptoms is 0.6% more likely to attempt suicide than that of those without depressive symptoms, and this probability increases to 4.2% among those with strong depressive symptoms. When considering sex and the presence of depressive symptoms, a Mexican adult woman has a higher probability of attempting suicide at some point in her life than a man, and the stronger the depressive symptoms are, the stronger the marginal effect between the sexes will be. In other words, the probability of a woman with strong depressive symptoms to attempt suicide is 2.7% higher than that of a man with the same level of depression. The probability of lifetime suicide attempt of a Mexican adult without depressive symptoms is 1.1% for women and 0.3% for men.

**Table 5:** Probability and marginal effects of lifetime suicide attempt among adults by sex and depressive symptoms in 2018 in Mexico.

Characteristics			Probability (%)	Marginal effect (%)
Sex		Males	0.6*	
		Females	1.6*	1.0*
Depression symptoms		Without symptoms	0.6*	
		Mild or moderate symptoms	1.2*	0.6*
		Strong symptoms	4.2*	3.6*
Sex and depression symptoms				
	Without symptoms	Males†	0.3*	
		Females	1.1*	0.8*
	Mild or moderate symptoms	Males†	1.1*	
		Females	1.6*	0.7‡
	Strong symptoms	Males†	2.9*	
		Females	5.6*	2.7*

n: 42,269.

* p-value<0.0001

† Reference category

‡ p-value<0.01.

## DISCUSSION

Suicidal behavior is a public health problem because of its prevalence and negative consequences^1^. We present an analysis of the prevalence of lifetime suicide attempt and its associated factors in Mexico. We found that the prevalence of suicide attempts among adults was 2% at the national level, which was found to be higher among women than among men and higher among young people than among older adults. These results remained after controlling for the effect of other variables. Low education was also associated with a higher prevalence of suicide attempts, whereas being married or cohabiting was a protective factor. Individuals clinically diagnosed with obesity, who have consumed or consume alcohol, or who were tobacco smokers at some point in their lives had a higher probability of attempting suicide than their counterparts without these characteristics. In terms of depression, Mexican adults with mild or moderate symptoms were 0.6% more likely to have attempted suicide at some time in their lives than those without depressive symptoms, and this probability increased to 4.2% among adults with strong symptoms.

Previous studies have reported that between 2.5 and 4.3% of Mexicans have attempted suicide at some time in their lives^9^. In this study, the prevalence of suicide attempts was lower. In another study, the authors found that the annual prevalence was 0.7%^10^. We also showed that women are more likely than men to attempt suicide, which is one of the most consistent results with previous studies on suicide^9,24^ because women attempt suicide more often than men, whereas men show higher levels of lethality in their attempts^25^. Female suicide attempts are even two to three times more frequent than male suicide attempts^11^, in line with the "gender paradox in suicide"^10^. Previous studies have suggested that differences in the way of expressing emotions or sociocultural gender norms related to suicidal behavior may explain why women are more likely to attempt suicide^26^. Committing suicide using lethal methods, and especially firearms, has been suggested as a sign of masculinity, and this factor may explain why suicide mortality is higher in men than in women^25^. The suicidal process, that is, the time from suicidal ideation to suicide attempt, is longer in women than in men, possibly due to gender differences in the propensity to use more lethal methods^27^.

As in other studies, the prevalence of suicide attempts was higher among younger people^11,28,29^. Similarly, the results showed that people with lower educational levels have a higher propensity to attempt suicide. Despite the lack of consensus on the association between education level and the risk of suicide attempt^30^, the results presented here corroborate previous studies^14,31-33^. A low education level is likely a marker of social disadvantage, which in turn is associated with various life stressors, including financial stress^11^, all of which are well-established associated factors^34^. A higher education level may lower the risk of suicide, possibly because of its effect on reducing the risk of depression and depressive symptoms^35^. Similarly, people who declared being in a relationship had a lower propensity to attempt suicide. This finding is similar to those of studies conducted in other countries, in which not being married or, more specifically, being divorced or experiencing a family breakdown was repeatedly reported as an associated factor for attempting suicide^36-38^.

Obesity, alcohol consumption, and tobacco smoking were positively associated with suicide attempt. The relationship between obesity and suicide has been previously observed^39^. This association has been explained by the complex interplay of factors in behavioral psychology and physiology. Body dissatisfaction and repeated failed attempts to lose weight, feelings of shame, and low self-esteem caused by the associated stigma of obesity may be associated with an increased risk of suicide^40^. Another factor is physical activity and its relationship with obesity, considering that inactive individuals more often have suicidal thoughts and are more likely to attempt suicide^41^. Alcohol consumption has also been reported as one of the main associated factors for suicidal behavior^42^. This association may be related to the effects of alcohol such as impulsivity, mood changes, and altered cognitive processes^30^. Alcohol consumption may also be related to the presence of psychiatric disorders that increase suicide attempts^43^. Similarly, tobacco smoking has been identified as an associated factor for suicidal behavior regardless of prior psychiatric or medical diagnoses or treatment^38,44^ possibly because addictive behaviors tend to affect people with a lower perception of danger, who are more impulsive and more likely to engage in life-threatening behaviors and who tend to act rashly^45^.

Depressive disorder played a key role in suicide attempts for both men and women, in line with previous studies^11,46,47^. Prior research has shown that 90% of people who died by suicide had one or more psychiatric disorders, particularly major depressive disorder, which accounted for between 59 and 87% of all suicides. This result implies that depression is a highly associated factor for suicidal behavior and a warning sign for completed suicide; in fact, depression has been established as the strongest predictor of such behavior^47^. Researchers have tried to explain the association between depression and suicide in different ways, including the biomechanisms of the nervous system^43^. Alternatively, for people with depression, the experience of discrimination and the social stigma of the disease may lead to suicide by increasing loneliness, hopelessness, and secrecy and by reducing self-esteem^48^.

Suicide attempts are an indicator of persistent mental health problems and a high risk of completed suicide, and of physical illnesses later in life^49^. Life expectancy is shortened by 18 years in men and by 11 years in women who first attempt suicide at 20 years of age^7^. A key measure of suicide prevention efforts consists of identifying the factors that influence the possibility of suicide attempts^18^. Approximately half of suicide deaths do not occur during the first attempt^50^. Therefore, preventing non-fatal attempts enables early interventions in a considerable number of people at a high risk of suicide and reduces the public health burden of suicidal behavior^14^. Prevention programs must include prevention strategies aimed at the entire population through actions such as reducing harmful alcohol consumption or tobacco smoking and vulnerable groups such as young people or people with mental health problems^1^.

The results from this study must be interpreted cautiously in light of some major limitations. First, the analysis was based on retrospective self-reported data, using face-to-face interviews which could be subject to underreporting and biased recall. Due to the social stigma associated with suicidal behavior and mental disorders, the prevalence rates of suicide attempts and depression may be underestimated^51^. Thus, our estimations should be considered a conservative estimate of the prevalence of suicide attempts. Second, although a high number of factors associated with suicide attempts were examined, some could not be included in this study, such as previous suicide attempts (violent or non-violent), because those who attempt suicide were highly likely to have made at least one previous attempt^12,52,53^. Third, our study had limitations in assessing intricate patterns of comorbidities, especially mental disorders which are highly comorbid. Understanding how comorbidity influences suicide attempts is crucial for gaining insights into the mechanisms by which associated factors, particularly mental disorders, contribute to suicidal behavior^54^. Fourth, since this was a cross-sectional study, we were unable to consider the temporality and order in which the associated factors were analyzed and the suicide attempts occurred (e.g., developing depression prior to suicide attempt), thereby precluding us from identifying causal relationships between them^55^. Fifth, the retrospective memory of adversity and traumatic life events, such as rape or sexual abuse, could not be included due to the small number of respondents who declared experiencing such events.

Research into suicide attempts is essential because of their psychological burden, impact, and the injuries they cause and their strong relationship with completed suicide. We found that the prevalence of suicide attempt was higher among women and young people. Regarding the associated factors, we found that low education, being single, having a diagnosis of obesity, consumption of alcohol or tobacco smoking, and having depression were associated with a higher prevalence of suicide attempts. These findings may assist clinicians in identifying patients with the highest likelihood of future suicide attempts. Identifying these factors is crucial for ensuring that individuals facing challenging situations do not view suicidal behavior as a viable option. Suicide prevention efforts should be strengthened by offering education and resources to individuals and families, equipping them with the tools to effectively manage and navigate conflicts. Timely assessment of the risk of suicide attempts becomes essential to help reduce mortality from suicide in the country^9^.

## Data Availability

The data that support the findings of this study are publicly available at https://ensanut.insp.mx/.

## References

[1] World Health Organization (2022). World mental health report: transforming mental health for all.

[2] Cerel J, Brown MM, Maple M, Singleton M, van de Venne J, Moore M (2019). How many people are exposed to suicide? Not six. Suicide Life Threat Behav.

[3] Pereira AS, Willhelm AR, Koller SH, Almeida RMM (2018). Risk and protective factors for suicide attempt in emerging adulthood. Cien Saude Colet.

[4] Valdez-Santiago R, Solórzano EH, Iñiguez MM, Burgos LA, Hernández HG, González AM (2018). Attempted suicide among adolescents in Mexico: prevalence and associated factors at the national level. Inj Prev.

[5] Sudol K, Mann JJ (2017). Biomarkers of suicide attempt behavior: towards a biological model of risk. Curr Psychiatry Rep.

[6] Kessler RC, Borges G, Walters EE (1999). Prevalence of and risk factors for lifetime suicide attempts in the National Comorbidity Survey. Arch Gen Psychiatry.

[7] Jokinen J, Talbäck M, Feychting M, Ahlbom A, Ljung R (2018). Life expectancy after the first suicide attempt. Acta Psychiatr Scand.

[8] Borges G, Orozco R, Benjet C, Medina-Mora ME (2010). Suicide and suicidal behaviors in Mexico: Retrospective and current status. Salud Publica Mex.

[9] Borges G, Orozco R, Mora MEM (2012). Risk index for attempted suicide in Mexico. Salud Pública Méx.

[10] Borges G, Orozco R, Villatoro J, Medina-Mora ME, Fleiz C, Díaz-Salazar J (2019). Suicide ideation and behavior in Mexico: Encodat 2016. Salud Pública Méx.

[11] Indu PS, Anilkumar TV, Pisharody R, Russell PS, Raju D, Sarma PS (2017). Prevalence of depression and past suicide attempt in primary care. Asian J Psychiatr.

[12] Shakeri J, Farnia V, Abdoli N, Akrami MR, Arman F, Shakeri H (2015). The risk of repetition of attempted suicide among Iranian women with psychiatric disorders as quantified by the suicide behaviors questionnaire. Oman Med J.

[13] Quinn PD, Fromme K (2010). Self-regulation as a protective factor against risky drinking and sexual behavior. Psychol Addict Behav.

[14] García de la Garza A, Blanco C, Olfson M, Wall MM (2021). Identification of suicide attempt risk factors in a national US survey using machine learning. JAMA Psychiatry.

[15] Mogensen H, Möller J, Hultin H, Mittendorfer-Rutz E (2016). Death of a close relative and the risk of suicide in Sweden-a large scale register-based case-crossover study. PLoS One.

[16] Fjeldsted R, Teasdale TW, Jensen M, Erlangsen A (2017). Suicide in relation to the experience of stressful life events: a population-based study. Arch Suicide Res.

[17] Aschan L, Goodwin L, Cross S, Moran P, Hotopf M, Hatch SL (2013). Suicidal behaviours in South East London: prevalence, risk factors and the role of socio-economic status. J Affect Disord.

[18] Liu BP, Zhang J, Chu J, Qiu HM, Jia CX, Hennessy DA (2019). Negative life events as triggers on suicide attempt in rural China: a case-crossover study. Psychiatry Res.

[19] Shamah-Levy T, Vielma-Orozco E, Heredia-Hernández O, Romero-Martínez M, Mojica-Cuevas J, Cuevas-Nasu L (2020). Encuesta Nacional de Salud y Nutrición 2018-19: resultados nacionales. Cuernavaca. México: Instituto Nacional de Salud Pública;.

[20] Royston P, Moons KGM, Altman DG, Vergouwe Y (2009). Prognosis and prognostic research: developing a prognostic model. BMJ.

[21] Chowdhury MZI, Turin TC (2020). Variable selection strategies and its importance in clinical prediction modelling. Fam Med Community Health.

[22] Coutinho LMS, Scazufca M, Menezes PR (2008). Métodos para estimar razão de prevalência em estudos de corte transversa. Rev Saúde Pública.

[23] Francisco PMSB, Donalisio MR, Azevedo MBA, Cesar CLG, Carandina L, Goldbaum M (2008). Medidas de associação em estudo transversal com delineamento complexo: razão de chances e razão de prevalência. Rev Bras Epidemiol.

[24] Vijayakumar L (2015). Suicide in women. Indian J Psychiatry.

[25] Bommersbach TJ, Rosenheck RA, Petrakis IL, Rhee TG (2022). Why are women more likely to attempt suicide than men? Analysis of lifetime suicide attempts among US adults in a nationally representative sample. J Affect Disord.

[26] Michaud L, Brovelli S, Bourquin C (2021). The gender paradox in suicide: some explanations and much uncertainty. Rev Med Suisse.

[27] Schrijvers DL, Bollen J, Sabbe BG (2012). The gender paradox in suicidal behavior and its impact on the suicidal process. J Affect Disord.

[28] Fakhari A, Farahbakhsh M, Esmaeili ED, Azizi H (2021). A longitudinal study of suicide and suicide attempt in northwest of Iran: incidence, predictors, and socioeconomic status and the role of sociocultural status. BMC Public Health.

[29] Parra-Uribe I, Blasco-Fontecilla H, Garcia-Parés G, Mártínez-Naval L, Valero-Coppin O, Cebrià-Meca A (2017). Risk of re-attempts and suicide death after a suicide attempt: a survival analysis. BMC Psychiatry.

[30] Dambrauskiene K, Adomaitiene V, Zalinkevicius R, Jariene G, Vilkas V, Rybakova I (2019). Can suicide attempt be related to problem drinking: cohort study. Alcohol Alcohol.

[31] Indu PV, Remadevi S, Vidhukumar K, Navas PMS, Anilkumar TV, Subha N (2020). Domestic violence as a risk factor for attempted suicide in married women. J Interpers Violence.

[32] Bálint L, Osváth P, Rihmer Z, Döme P. (2016). Associations between marital and educational status and risk of completed suicide in Hungary. J Affect Disord.

[33] Mackenbach JP, Kulhánová I, Bopp M, Deboosere P, Eikemo TA, Hoffmann R (2015). Variations in the relation between education and cause-specific mortality in 19 European populations: a test of the "fundamental causes" theory of social inequalities in health. Soc Sci Med.

[34] Knipe DW, Carroll R, Thomas KH, Pease A, Gunnell D, Metcalfe C (2015). Association of socio-economic position and suicide/attempted suicide in low and middle income countries in South and South-East Asia-a systematic review. BMC Public Health.

[35] Assari S, Schatten HT, Arias AS, Miller IW, Camargo CA, Boudreaux ED (2019). Higher educational attainment is associated with lower risk of a future suicide attempt among non-hispanic whites but not non-hispanic blacks. J Racial Ethn Health Disparities.

[36] Kim YT, Cha C, Lee MR (2021). Comparison of causes for suicidal ideation and attempt: Korean Longitudinal Survey of Women and Families. Arch Womens Ment Health.

[37] Næss EO, M ehlum L, Qin P (2021). Marital status and suicide risk: temporal effect of marital breakdown and contextual difference by socioeconomic status. SSM Popul Health.

[38] Echeverria I, Cotaina M, Jovani A, Mora R, Haro G, Benito A (2021). Proposal for the inclusion of tobacco use in suicide risk scales: results of a meta-analysis. Int J Environ Res Public Health..

[39] Branco JC, Motta J, Weiner C, Oses JP, Moreira FP, Spessato B (2017). Association between obesity and suicide in woman, but not in man: a population-based study of young adults. Psychol Health Med.

[40] Wagner B, Klinitzke G, Brähler E, Kersting A (2013). Extreme obesity is associated with suicidal behavior and suicide attempts in adults: results of a population-based representative sample. Depress Anxiety.

[41] Schneider B, Lukaschek K, Baumert J, Meisinger C, Erazo N, Ladwig KH (2014). Living alone, obesity, and smoking increase risk for suicide independently of depressive mood findings from the population-based MONICA/KORA Augsburg cohort study. J Affect Disord.

[42] Darvishi N, Farhadi M, Haghtalab T, Jalal P (2015). Alcohol-related risk of suicidal ideation, suicide attempt, and completed suicide: a meta-analysis. PLoS One.

[43] Choi SB, Lee W, Yoon JH, Won JU, Kim DW (2017). Risk factors of suicide attempt among people with suicidal ideation in South Korea: a cross-sectional study. BMC Public Health.

[44] Evins AE, Korhonen T, Kinnunen TH, Kaprio J (2017). Prospective association between tobacco smoking and death by suicide: A competing risks hazard analysis in a large twin cohort with 35-year follow-up. Psychol Med.

[45] Thomsen KR, Callesen MB, Hesse M, Kvamme TL, Pedersen MM, Pedersen MU (2018). Impulsivity traits and addiction-related behaviors in youth. J Behav Addict.

[46] Mayer L, Rüsch N, Frey LM, Nadorff MR, Drapeau CW, Sheehn L (2020). Anticipated suicide stigma, secrecy, and suicidality among suicide attempt survivors. Suicide Life Threat Behav.

[47] Dong M, Zeng LN, Lu L, Li XH, Ungvari GS, Ng CH (2019). Prevalence of suicide attempt in individuals with major depressive disorder: a meta-analysis of observational surveys. Psychol Med.

[48] Oexle N, Herrmann K, Staiger T, Sheehan L, Rüsch N, Krumm S (2019). Stigma and suicidality among suicide attempt survivors: a qualitative study. Death Stud.

[49] Goldman-Mellor SJ, Caspi A, Harrington H, Hogan S, Nada-Raja S, Poulton R (2014). Suicide attempt in young people: a signal for long-term health care and social needs. JAMA Psychiatry.

[50] Bostwick JM, Pabbati C, Geske JR, McKean AJ (2016). Suicide attempt as a risk factor for completed suicide: even more lethal than we knew. Am J Psychiatry.

[51] Naghavi M, Global Burden of Disease Self-Harm Collaborators (2019). Global, regional, and national burden of suicide mortality 1990 to 2016: systematic analysis for the Global Burden of Disease Study 2016. BMJ.

[52] Mullen D, Hendren R, Parmelee D, David RB (2018). El niño o adolescente suicida.

[53] Haukka J, Suominen K, Partonen T, Lönnqvist J (2008). Determinants and outcomes of serious attempted suicide: a nationwide study in Finland, 1996-2003. Am J Epidemiol.

[54] Nock M, Hwang I, Sampson NA, Kessler RC (2010). Mental disorders, comorbidity and suicidal behavior: results from the National Comorbidity Survey Replication. Mol Psychiatry.

[55] Pérez-Amezcua B, Rivera-Rivera L, Atienzo EE, Castro F, Leyva-López A, Chávez-Ayala R (2010). Prevalencia y factores asociados a la ideación e intento suicida en adolescentes de educación media superior de la República Mexicana. Salud Publica Mex.

